# Supplementary material for: Nutritional control of body size through FoxO-Ultraspiracle mediated ecdysone biosynthesis
Source: eLife. 2014 Nov 25;3:e03091. doi: 10.7554/eLife.03091 (PMC4337420; doi:10.7554/eLife.03091)
Supplement: Supplementary file 1. — Means for age and size at critical weight and time to metamorphosis from critical weight ±95% confidence intervals. Statistical comparisons for age at critical weight and size at critical weight between genotypes and treatments are shown in Figures 3, 6 and Figure 6—figure supplement 2. Genotypes fed on ecdysone-supplemented medium (+20E) do not show delays in development and therefore age at critical weight cannot be determined (na). For time from critical weight to pupariation, mean times to pupariation within the same shaded box that share the same letter are statistically indistinguishable. Those that differ in letter are significantly different, as determined by Permutation tests (p < 0.034). DOI: http://dx.doi.org/10.7554/eLife.03091.019 [file elife03091s001.docx]

| Background | Genotype | Age at critical weight  (h AL3E) | | | Size at critical weight  (mg) | | | Time from critical weight to pupariation | | |
| --- | --- | --- | --- | --- | --- | --- | --- | --- | --- | --- |
|  |  | Age | 95% - | 95% + | Size | 95% - | 95% + | Time | 95% - | 95% + |
| *FoxO wild type* | *w^1118^* | 8.66 | 6.00 | 0.75 | 0.62 | 0.86 | 10.66 | 42.26^a^ | 37.51 | 48.04 |
|  | *w^1118^ +*20E | na | na | na | na | na | na | 32.79^a^ | 30.79 | 34.22 |
|  | *Phm*>+ | 8.44 | 8.13 | 0.74 | 0.72^a^ | 0.83 | 8.75 | 44.70^a^ | 44.70 | 44.70 |
|  | *ND*>FoxO | 8.26 | 8.06 | 0.69 | 0.67^a^ | 0.79 | 8.46 | 45.87^a^ | 45.87 | 45.87 |
|  | *Phm*>FoxO | 19.11 | 18.08 | 1.35 | 1.30^b^ | 1.41 | 20.13 | 33.54^b^ | 33.53 | 33.55 |
|  | *Phm*>FoxO NK | 13.04 | 12.08 | 1.04 | 0.98 | 1.15 | 13.99 | 43.65^c^ | 43.64 | 43.65 |
|  | *ND*>Usp | 8.20 | 8.08 | 0.67 | 0.65 | 0.69 | 8.32 | 43.34^a^ | 43.33 | 43.34 |
|  | *Phm*>Usp | 10.59 | 10.22 | 0.74 | 0.71 | 0.77 | 10.97 | 51.27^a^ | 51.26 | 51.27 |
|  | *ND*>FoxO, Usp | 8.23 | 6.00 | 0.70 | 0.61 | 0.73 | 8.84 | 46.50^a^ | 44.40 | 49.29 |
|  | *Phm*>FoxO, Usp | 21.44 | 16.00 | 1.70 | 1.42 | 1.80 | 23.20 | 39.29^b^ | 31.21 | 59.89 |
|  | *ND*>dsFoxO | 10.07 | 9.52 | 0.78 | 0.70 | 0.81 | 10.62 | 39.51^a^ | 39.50 | 39.51 |
|  | *Phm*>dsFoxO | 6.27 | 6.13 | 0.52 | 0.50 | 0.54 | 6.43 | 48.99^a^ | 47.62 | 50.45 |
|  | *ND*>dsUsp | 10.85 | 9.69 | 0.75 | 0.72 | 0.81 | 12.01 | 39.19^a^ | 39.18 | 39.20 |
|  | *Phm*>dsUsp | 8.26 | 7.59 | 0.43 | 0.39 | 0.45 | 8.92 | 48.82^a^ | 48.81 | 48.82 |
|  | *ND*>dsFoxO, dsUsp | 10.87 | 8.44 | 0.87 | 0.75 | 0.96 | 2.68 | 38.56^a^ | 34.31 | 44.05 |
|  | *Phm*>dsFoxO, dsUsp | 4.73 | 4.40 | 0.34 | 0.32 | 0.35 | 5.15 | 49.63^b^ | 48.32 | 51.01 |
| *in FoxOD94/Df* | *P0206*>+ +20E | na | na | na | na | na | na | 36.54^a^ | 34.54 | 38.18 |
|  | *P0206*>FoxO + 20E | na | na | na | na | na | na | 56.72^b^ | 54.75 | 58.66 |
|  | *P0206*>+ | 11.21 | 10.64 | 0.81 | 0.75 | 0.94 | 11.78 | 43.90^a^ | 43.89 | 43.90 |
|  | *ND*>FoxO | 10.53 | 10.26 | 0.76 | 0.71 | 0.81 | 10.80 | 45.29^a^ | 45.29 | 45.30 |
|  | *P0206*>FoxO | 30.15 | 24.31 | 1.77 | 1.49 | 1.93 | 33.02 | 32.66^b^ | 26.01 | 51.25 |
|  | *P0206*>FoxO NK | 20.59 | 19.99 | 1.29 | 1.19 | 1.44 | 21.20 | 44.54^a^ | 44.54 | 44.55 |
|  | *ND*>FoxO NK, Usp | 10.30 | 8.00 | 0.75 | 0.64 | 0.79 | 11.06 | 45.64^a^ | 43.24 | 48.33 |
|  | *P0206*>FoxO NK, Usp | 20.43 | 18.61 | 1.14 | 1.06 | 1.26 | 23.27 | 57.11^b^ | 48.01 | 62.48 |

**Supplementary Table 1: Means for age and size at critical weight and time to metamorphosis from critical weight ± 95% confidence intervals.** Statistical comparisons for age at critical weight and size at critical weight between genotypes and treatments are shown in Figures 3, 6 and 6-figure supplement 2. Genotypes fed on ecdysone-supplemented medium (+ 20E) do not show delays in development and therefore age at critical weight cannot be determined (na). For time from critical weight to pupariation, mean times to pupariation within the same shaded box that share the same letter are statistically indistinguishable. Those that differ in letter are significantly different, as determined by Permutation tests (p<0.034).
