# Supplementary material for: Nutritional control of body size through FoxO-Ultraspiracle mediated ecdysone biosynthesis
Source: eLife. 2014 Nov 25;3:e03091. doi: 10.7554/eLife.03091 (PMC4337420; doi:10.7554/eLife.03091)
Supplement: Supplementary file 2. — Primers used for quantitative PCR. DOI: http://dx.doi.org/10.7554/eLife.03091.020 [file elife03091s002.docx]

| Gene product |  | Primer |
| --- | --- | --- |
| *RpL3* | Forward | AAGGATGACGCCAGCAAGCCAGTC |
|  | Reverse | TAGCCGACAGCACCGACCACAATC |
| *foxo* | Forward | AGCAACCTCAGCAACATAAGCAG |
|  | Reverse | TCAGATTTGTGGTAGCCGTTTGTG |
| *usp* | Forward | GAGCTGAGTGTAAAGATGAAGCGG |
|  | Reverse | GTAATGCGGAAGAGGAACAGGTG |
| *phm* | Forward | ATGCAAAGAGAACTTCGTCGTGGG |
|  | Reverse | GACCCGTAAAGAGCGTGAGTATC |
| *dib* | Forward | GTAGTGCCCTCAATCCCTATCTG |
|  | Reverse | TCTTCACACCCATCTCATCATCGG |
| *e74B* | Forward | ATGGGCAGCAGGCTAAGACTCAG |
|  | Reverse | TACGGATTCAGACTCCTCTTCATC |

**Supplementary Table S2: Primers used for quantitative PCR.**
