# Supplementary material for: Nutritional control of body size through FoxO-Ultraspiracle mediated ecdysone biosynthesis
Source: eLife. 2014 Nov 25;3:e03091. doi: 10.7554/eLife.03091 (PMC4337420; doi:10.7554/eLife.03091)
Supplement: Supplementary file 3. — Primers used for Luciferase constructs. DOI: http://dx.doi.org/10.7554/eLife.03091.021 [file elife03091s003.docx]

| Promoter |  | Primer |
| --- | --- | --- |
| *inr* | Forward | AGATCTACACAAACGTGTGCGTGAGAGAG (*Bam*HI site included) |
|  | Reverse | GCGGCCGCGCATATTGCGGTGTTTCTATTC (*Not*I site included) |
| *4e-bp* | Forward | AGATCTCAAATATGCTCCTCGTCACATGG (*Bam*HI site included) |
|  | Reverse | GCGGCCGCGCATCTTAGCTGATTGATTGGATTGG (*Not*I site included) |

**Supplementary Table S3: Primers used for Luciferase constructs.**
